# TMEM16A and TMEM16B Modulate Pheromone-Evoked Action Potential Firing in Mouse Vomeronasal Sensory Neurons

**DOI:** 10.1523/ENEURO.0179-21.2021

**Published:** 2021-09-15

**Authors:** Andres Hernandez-Clavijo, Nicole Sarno, Kevin Y. Gonzalez-Velandia, Rudolf Degen, David Fleck, Jason R. Rock, Marc Spehr, Anna Menini, Simone Pifferi

**Affiliations:** 1 Neurobiology Group, SISSA, International School for Advanced Studies, Trieste 34136, Italy; 2 Department of Chemosensation, Institute for Biology II, RWTH Aachen University, Aachen D-52074, Germany; 3 Center for Regenerative Medicine, Boston University School of Medicine, Boston 02118, MA; 4 Department of Experimental and Clinical Medicine, Università Politecnica delle Marche, Ancona 60126, Italy

**Keywords:** ion channel, sensory, TMEM16, vomeronasal

## Abstract

The mouse vomeronasal system controls several social behaviors. Pheromones and other social cues are detected by sensory neurons in the vomeronasal organ (VNO). Stimuli activate a transduction cascade that leads to membrane potential depolarization, increase in cytosolic Ca^2+^ level, and increased firing. The Ca^2+^-activated chloride channels TMEM16A and TMEM16B are co-expressed within microvilli of vomeronasal neurons, but their physiological role remains elusive. Here, we investigate the contribution of each of these channels to vomeronasal neuron firing activity by comparing wild-type (WT) and knock-out (KO) mice. Performing loose-patch recordings from neurons in acute VNO slices, we show that spontaneous activity is modified by *Tmem16a* KO, indicating that TMEM16A, but not TMEM16B, is active under basal conditions. Upon exposure to diluted urine, a rich source of mouse pheromones, we observe significant changes in activity. Vomeronasal sensory neurons (VSNs) from *Tmem16a* cKO and *Tmem16b* KO mice show shorter interspike intervals (ISIs) compared with WT mice, indicating that both TMEM16A and TMEM16B modulate the firing pattern of pheromone-evoked activity in VSNs.

## Significance Statement

Vomeronasal sensory neurons (VSNs) express two Ca^2+^-activated chloride channels TMEM16A and TMEM16B, however their physiological role is still unclear. Using a loss of function approach, we found that TMEM16A modulates the pattern of VSN spontaneous spike activity, while TMEM16A and TMEM16B reduced the instant frequency of pheromone-evoked activity. These new findings call for a reconsideration of the patterns of the peripheral coding of sensory stimuli.

## Introduction

Most mammals use at least two distinct olfactory systems to detect chemicals: the main and the accessory (vomeronasal) olfactory system. In general, pheromones affect the physiology and/or behavior of an individual by activating either one or both systems (Tirindelli et al., 2009; Touhara and Vosshall, 2009; Mohrhardt et al., 2018).

Chemical signals bind to chemosensory neurons in the vomeronasal organ (VNO), a bilateral cylindrical structure within a cartilaginous capsule located beneath the septum (Døving and Trotier, 1998). Vomeronasal sensory neurons (VSNs) are bipolar neurons with a single apical dendrite that has a terminal swelling (knob) with several microvilli, where vomeronasal receptors (V1Rs, V2Rs, FPRs) are located and sensory transduction occurs. Each VSN expresses one type or few types of vomeronasal receptors, and axons from VSNs expressing the same receptor project to various glomeruli in the accessory olfactory bulb (AOB; Rodriguez et al., 1999; Francia et al., 2014).

Vomeronasal receptor-ligand binding activates a G-protein mediated signaling pathway involving phospholipase C activation. Phosphoinositide turnover activates transient receptor potential canonical 2 (TRPC2) channels allowing the influx of Na^+^ and Ca^2+^ (Liman et al., 1999; Lucas et al., 2003; Leinders-Zufall et al., 2018). In parallel, Ca^2+^ release from intracellular stores has also been described (Kim et al., 2011). Ultimately, VSN signal transduction generates membrane depolarization and action potentials that are sent to the AOB.

The increase in intracellular Ca^2+^ concentration on activation of the transduction cascade has several physiological effects, including the activation of Ca^2+^-activated chloride channels (Yang and Delay, 2010; Kim et al., 2011; Dibattista et al., 2012; Amjad et al., 2015). At least two members of the TMEM16 family, TMEM16A and TMEM16B, form Ca^2+^-activated chloride channels and are expressed in several different tissues (Caputo et al., 2008; Schroeder et al., 2008; Yang et al., 2008; Pifferi et al., 2009a; Stephan et al., 2009; Pedemonte and Galietta, 2014). Interestingly, although TMEM16A and TMEM16B are individually expressed in different cell types, they co-express in microvilli of VSNs (Dibattista et al., 2012; Münch et al., 2018). Not many other cell types are known to co-express TMEM16A and TMEM16B. Indeed, to our knowledge, the only other cells that have been shown to functionally express both proteins are pinealocytes (Yamamura et al., 2018). Therefore, studying the role of these channels in the VNO is helpful to understand how VSNs respond to pheromones and contributes to understanding their individual role in other cells co-expressing them.

In isolated VSNs, Amjad et al. (2015) have shown that the biophysical properties of Ca^2+^-activated chloride currents, measured both in whole-cell and in inside-out patches from the apical neuronal region, resemble those of TMEM16A, rather than TMEM16B channels. Moreover, Ca^2+^-activated chloride currents were abolished in VSNs from *Tmem16a* conditional knock-out mice (cKO), demonstrating that TMEM16A is a necessary component of Ca^2+^-activated chloride channels in mouse VSNs (Amjad et al., 2015; Münch et al., 2018). Different results were obtained in *Tmem16b* KO mice, with a study reporting the lack of Ca^2+^-activated chloride currents in VSNs (Billig et al., 2011) and another one showing that disruption of *Tmem16b* only insignificantly reduced those currents (Münch et al., 2018).

In this study, we investigated the individual roles of TMEM16A and TMEM16B in controlling physiological VSN activity. We compared spontaneous and stimulus-induced firing in wild-type (WT), cKO for *Tmem16a* or mCherry *Tmem16b* KO (Zhang et al., 2017) mice. We found that only TMEM16A is involved in spontaneous firing patterns, while both TMEM16A and TMEM16B modulate the firing pattern of pheromone-evoked activity in VSNs.

## Materials and Methods

### Animals

Mice were handled in accordance with the Italian Guidelines for the Use of Laboratory Animals and European Union on animal research, under a protocol approved by the ethics committee of Scuola Internazionale Superiore di Studi Avanzati (SISSA) and approved by Italian Ministry of Health.

The generation of *Tmem16a* cKO mice has been previously described in detail (Amjad et al., 2015). *Tmem16a* cKO mice were homozygous for the floxed *Tmem16a* alleles and heterozygous for *Cre* and *Omp* (Li et al., 2004; Faria et al., 2014; Schreiber et al., 2015). *Tmem16a^fl/fl^* mice were used as controls.

mCherry *Tmem16b* KO mice were kindly provided by Lily Jan (University of California, San Francisco, Zhang et al., 2017) and were generated by inserting *mCherry* sequence with a farnesylation signal at the C terminus (mCherry-F) in frame with the alternative start ATG codon in the third exon of *Tmem16b*, therefore membrane associated mCherry marks neurons that normally express *Tmem16b* (Zhang et al., 2017; see their Fig. S2C).

V2r1b-tau-GFP mice were provided by Peter Mombaerts (Max Planck Research Unit Neurogenetics, Frankfurt, Germany; Rodriguez et al., 1999).

All experiments were performed on tissues from two to three months old mice of either sex. All experiments and data analysis were performed by researchers who were blind to the mouse genotype.

### Immunohistochemistry

VNO sections and immunohistochemistry were obtained as described previously (Dibattista et al., 2012; Amjad et al., 2015). The following primary antibodies were used: polyclonal goat anti-TMEM16A (1:200; sc-69 343, Santa Cruz), polyclonal rabbit anti-TMEM16B (1:500; NBP1-90739, Novus), rabbit anti-RFP (1:500; 600-401-379, Rockland), goat anti-OMP (1:1000; 544-1001, Wako). The following secondary antibodies obtained from Life Technologies (dilution; catalog number) were used: donkey anti-rabbit Alexa Fluor 594 Plus (1:500; A32754), donkey anti-goat Alexa Fluor 488 Plus (1:500; A32814). Immunoreactivity was visualized with a confocal laser scanning microscope (Nikon A1R). Images were acquired using NIS-Elements Nikon software (at 1024 × 1024-pixel resolution) and were not modified other than to balance brightness and contrast. Nuclei were stained by DAPI. Control experiments without the primary antibodies gave no signal.

### Clarity and imaging

Brains and VNOs from adult mice (V2R1b-GFP-TMEM16A^fl/fl^-OMP/OMP and V2R1b-GFP-TMEM16A^fl/fl^-OMP/Cre) of both sexes were dissected. Brains were further sliced in 3 mm coronal sections including the AOB. To maintain structural integrity, the tissue was fixed overnight at 4°C in hydrogel fixation solution containing 4% acrylamide, 0.05% bis-acrylamide, 0.25% VA-044 Initiator (Wako Chemicals), 4% PFA in PBS. After polymerization, lipids were removed by passive clearing. The samples were incubated at 37°C for two months in 4% SDS and 200 mm boric acid at pH 8.5. The buffer was changed every other week. During the last clearing cycle, the nuclear marker DRAQ5 (1:1000, Thermo Fisher Scientific) was added to each sample. After washing steps, samples were incubated for 24 h in RIMS80, containing 80 g Nycodenz (PROGEN Biotechnik), 20 mm PS (phosphate buffer, pH 7.5), 0.1% Tween 20, and 0.01% sodium azide. Imaging was performed using a Leica SP8 DLS microscope equipped with a 488 and 633 nm diode laser, an HC PL FLUOTAR 5×/0.15 IMM DLS observing objective, an L 1.6×/0.05 DLS illuminating objective and a DLS TwinFlect 7.8 mm Gly mirror cap that creates the lightsheet. Specimens were positioned in a custom-made 3D-printed chamber.

Data were analyzed using IMARIS 9.5.1 (Oxford Instruments). AOB glomerular volume was determined by measuring the volume of all fluorescently labeled glomerular structures within the AOB. VNO cell density was measured by counting the number of fluorescently labeled cells in a defined volume of vomeronasal sensory epithelium (VNE). MATLAB R2019b (MathWorks) was used for plotting and statistical analysis. CorelDRAW 2019 (Corel Corporation) was used for figure preparation.

### Preparation of acute slices of mouse VNO

Acute slices of mouse VNO were prepared as previously described (Shimazaki et al., 2006; Dibattista et al., 2008; Wong et al., 2018). In brief, the VNO was removed and transferred to ice-cold artificial CSF (ACSF) containing the following: 120 mm NaCl, 20 mm NaHCO_3_, 3 mm KCl, 2 mm CaCl_2_, 1 mm MgSO_4_, 10 mm HEPES, and 10 mm glucose, pH 7.4. The capsule and all cartilaginous tissues were carefully removed and the two halves of the VNO were isolated from the vomer bone. Each half of the VNO was then separately treated. The VNO was embedded in 3% Type I-A agarose (Sigma) prepared in ACSF once the agar had cooled to 38°C. Upon solidification, the agar block was fixed in a glass Petri dish and sliced with a vibratome (Vibratome 1000 Plus Sectioning System) at 200 to 250 μm thickness in oxygenated ACSF solution. Slices were then left to recover for >30 min in chilled and oxygenated ACSF before electrophysiological experiments were initiated.

### Electrophysiological recordings from VSNs and stimuli presentation

Slices were viewed with an upright microscope (Olympus BX51WI) by infrared differential contrast optics with water immersion 20× or 60× objectives. The slice preparation maintained the VNO cross-sectional structure and many individual VSNs could be clearly distinguished by their morphology. Whole-cell or loose-patch recordings were obtained using patch pipettes pulled from borosilicate capillaries (WPI) with a PC-10 puller (Narishige) with a resistance of 3–6 MΩ. Recordings were obtained with a Multiclamp 700B amplifier controlled by Clampex 10 via a Digidata 1440 (Molecular Devices). Data were low-pass filtered at 2 kHz and sampled at 10 kHz. Experiments were performed at room temperature (20–25°C). The recording chamber was continuously perfused with oxygenated (95% O_2_, and 5% CO_2_) ACSF by gravity flow. The slice was anchored to the base of the recording chamber using a homemade U-shaped silver wire, holding down the agar support without touching the slice itself.

Membrane properties and inward and outward voltage-gated currents were measured in the whole-cell configuration with a KCl based intracellular solution containing the following: 80 mm K-gluconate, 60 mm KCl, 10 mm NaCl, 1 mm MgCl_2_, 0.023 mm CaCl_2_, 10 mm HEPES, and 10 mm EGTA, adjusted to pH 7.2 with KOH.

For recordings of Ca^2+^-activated currents, we used a CsCl based intracellular solution containing the following: 140 mm CsCl, 10 mm HEPES, and 10 mm HEDTA, adjusted to pH 7.2 with CsOH. The nominally 0 Ca^2+^ solution contained no added Ca^2+^. The 1.5 μm free Ca^2+^ solution was obtained by adding 3.209 mm CaCl_2_. The added concentration was the same as that previously used to obtain 1.5 μm free Ca^2+^ experimentally determined by Fura-4F (Invitrogen) measurements by using a LS-50B luminescence spectrophotometer (PerkinElmer; Pifferi et al., 2006, 2009a,b) and based on calculation with the program WinMAXC (C. Patton, Stanford University, Stanford, CA; Patton et al., 2004).

Extracellular recordings from the soma of VSNs were obtained in the on-cell loose-patch configuration with seal resistances of 40–100 MΩ. Pipette solution was ACSF, as the bath solution, and the recordings were made in voltage-clamp mode with a holding potential of 0 mV.

Stimuli were focally delivered through a gravity-fed multivalve perfusion system (ALA-VM8, ALA Scientific Instruments). The tip of the perfusion head, with a diameter of 360 μm, was placed ∼500 μm away from the slice. The time delay of the stimulus was measured using a high-K^+^ ACSF solution. To avoid mechanical artifacts, the slice was constantly perfused with ACSF and the flow out of the pipette was switched between ACSF and stimulus solutions. This results in a constant flow across the epithelium and sharp concentration transients, undiluted by the bath ACSF.

### Urine collection and stimulus solutions

Urine was collected from both sexes C57BL/6 mice, filtered with a 0.2-μm filter, and frozen at −80°C. Before use, male and female urines were mixed in a 1:1 ratio, and the mixture was diluted to 1:50 in ACSF (pH 7.4). As mouse urine contains urea and K^+^, which could potentially cause neurons to fire by direct membrane depolarization, we used artificial urine diluted 1:50 in ACSF as a negative control. Artificial urine contained the following: 100 mm NaCl, 40 mm KCl, 20 mm NH_4_OH, 4 mm CaCl_2_, 2.5 mm MgCl_2_,15 mm NaH_2_PO_4_, 20 mm NaHSO_4_, and 333 mm urea, pH 7.4 adjusted with NaOH (Holy et al., 2000). To test cell viability, we used a high K^+^ solution (25 mm KCl) by replacing equimolar amounts of NaCl with KCl in ACSF. Diluted urine or artificial urine stimuli were applied in 10-s pulses with an interstimulus interval of at least 4 min to avoid desensitization or adaptation processes (Spehr et al., 2009; Wong et al., 2018). High K^+^ solution was applied in 5-s pulses. All compounds and chemicals were obtained from Sigma-Aldrich, unless otherwise stated.

### Analysis of electrophysiological data

IgorPro software (WaveMetrics) and Clampfit (Molecular Devices) were used for data analysis and figure preparation. In loose-patch, slow changes in the baseline were corrected by filtering the recordings offline with a high-pass filter at 2 Hz. Spikes were initially detected using an event detection tool using an arbitrary threshold, and then confirmed by shape inspection. For spontaneous activity, the mean firing activity was estimated by dividing the number of spontaneous spikes by the duration of recordings in the absence of stimuli. Interspike interval (ISI) was calculated by measuring the time between each consecutive spike. Each ISI distribution was obtained by grouping the ISIs in bins, as indicated in the figures, and normalized by dividing the value for each bin by the total number of ISIs. The values in *y*-axes represent the percentage of spikes in each bin and the area under the curve is 100%.

For VSNs showing a high spontaneous activity, it was difficult to identify a urine response from a single recording. To avoid false positives, we applied at least three repetitive stimulations. Each recording was 90 s long divided into 40 s of prestimulus, 10 s of stimulus, and 40 s of poststimulus. To analyze the response to urine for a single cell we took the basal period (40 s prestimulation) and stimulus period (10 s of stimulation), and we calculated the basal and the stimulation frequency for each trace. We defined a threshold level as: BF + 2*σ^2^, where BF is the average of basal frequency for all the traces and σ^2^ is the SD of the basal frequency. A cell was considered responsive to urine if: (1) the average spike frequency was above threshold during stimulation; and (2) there was no response to diluted artificial urine.

### Statistical analysis

Data are presented as mean ± SEM, with *n* indicating the number of neurons. VSNs were obtained from at least five (whole-cell recordings) or 10 (loose-patch recordings) different WT or mutant mice. Normal distribution of the data was tested with the Shapiro–Wilk test or with the Jarque–Bera test. For normally distributed data, statistical significance was determined using unpaired Student’s *t* tests. For multiple comparison of the data, we used Dunn–Hollander–Wolfe test after Kruskal–Wallis analysis. Kolmogorov–Smirnov test was used to compare cumulative distributions. *p*-values <0.05 were considered significant. Statistical analysis was performed with IgorPro software (Wavemetrics).

## Results

### TMEM16A and TMEM16B expression in the vomeronasal epithelium

**Figure 1.**
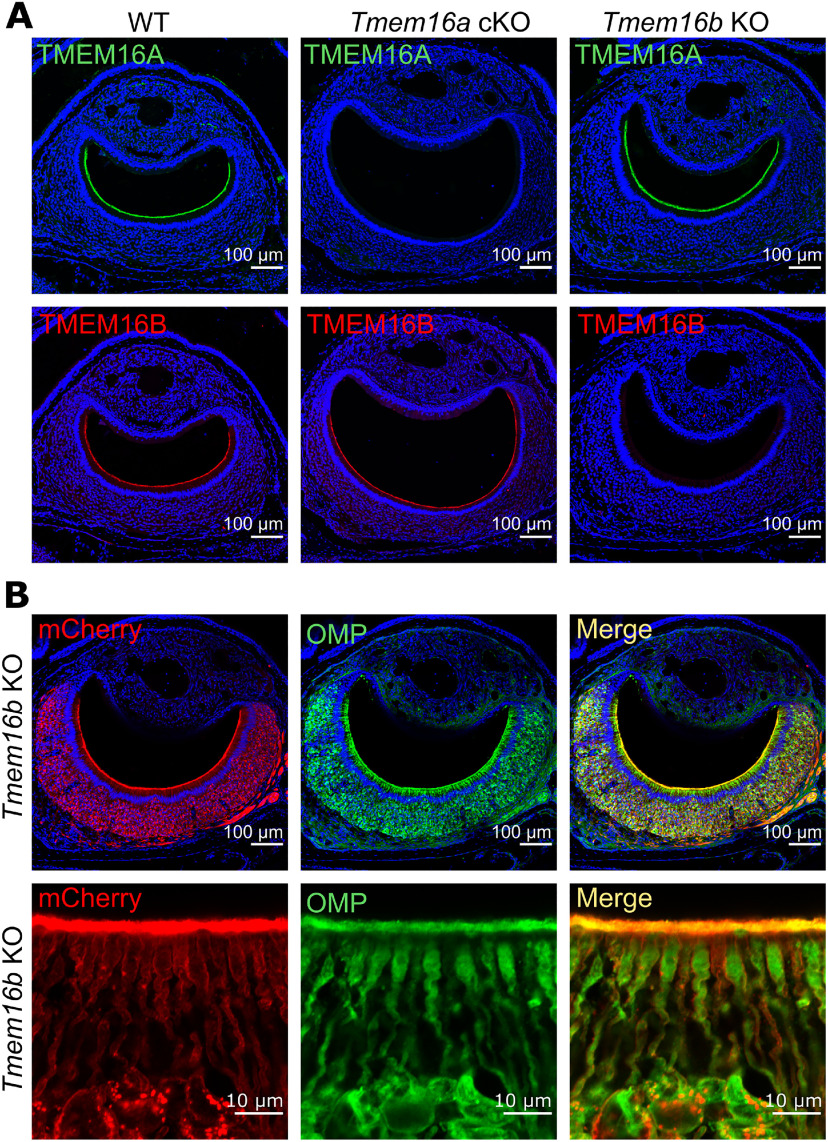
TMEM16A and TMEM16B are expressed at the apical surface of the vomeronasal epithelium. ***A***, Confocal micrographs of coronal sections of the VNO from WT, *Tmem16a* cKO, or *Tmem16b* KO mice, as indicated, immunostained with antibody against TMEM16A (green) and TMEM16B (red). No immunoreactivity to TMEM16A or TMEM16B was detectable in *Tmem16a* cKO or *Tmem16b* KO mice, respectively. ***B***, Confocal micrographs of VNO sections from *Tmem16b* KO mice expressing mCherry in the membrane of cells that normally express TMEM16B, immunostained with antibody against mCherry (red) and OMP (green). At the bottom, a magnification showing the co-expression of mCherry and OMP. Note that mCherry is expressed, as expected, in the membrane of entire VSNs and not only at the apical side. Cell nuclei were stained by DAPI (blue).

TMEM16A and TMEM16B are co-expressed in microvilli of mouse VSNs (Dibattista et al., 2012; Münch et al., 2018) but their functional role is unclear. By immunohistochemistry, we confirmed that TMEM16A and TMEM16B are expressed at the apical surface of the vomeronasal epithelium of WT mice (Fig. 1*A*), whereas TMEM16A or TMEM16B immunoreactivity was absent, as expected, in *Tmem16a* cKO and *Tmem16b KO* mice, respectively.

mCherry *Tmem16b* KO mice (Zhang et al., 2017) allow visualization of those cells that normally express TMEM16B by membrane staining with farnesylated mCherry. Figure 1*B* clearly shows co-expression of OMP and mCherry in VSNs, confirming the expression of TMEM16B in VSNs and excluding TMEM16B expression in supporting cells of the VNO.

### Ca^2+^-activated chloride currents in VSNs from WT, *Tmem16a* cKO, and *Tmem16b* KO mice

**Figure 2.**
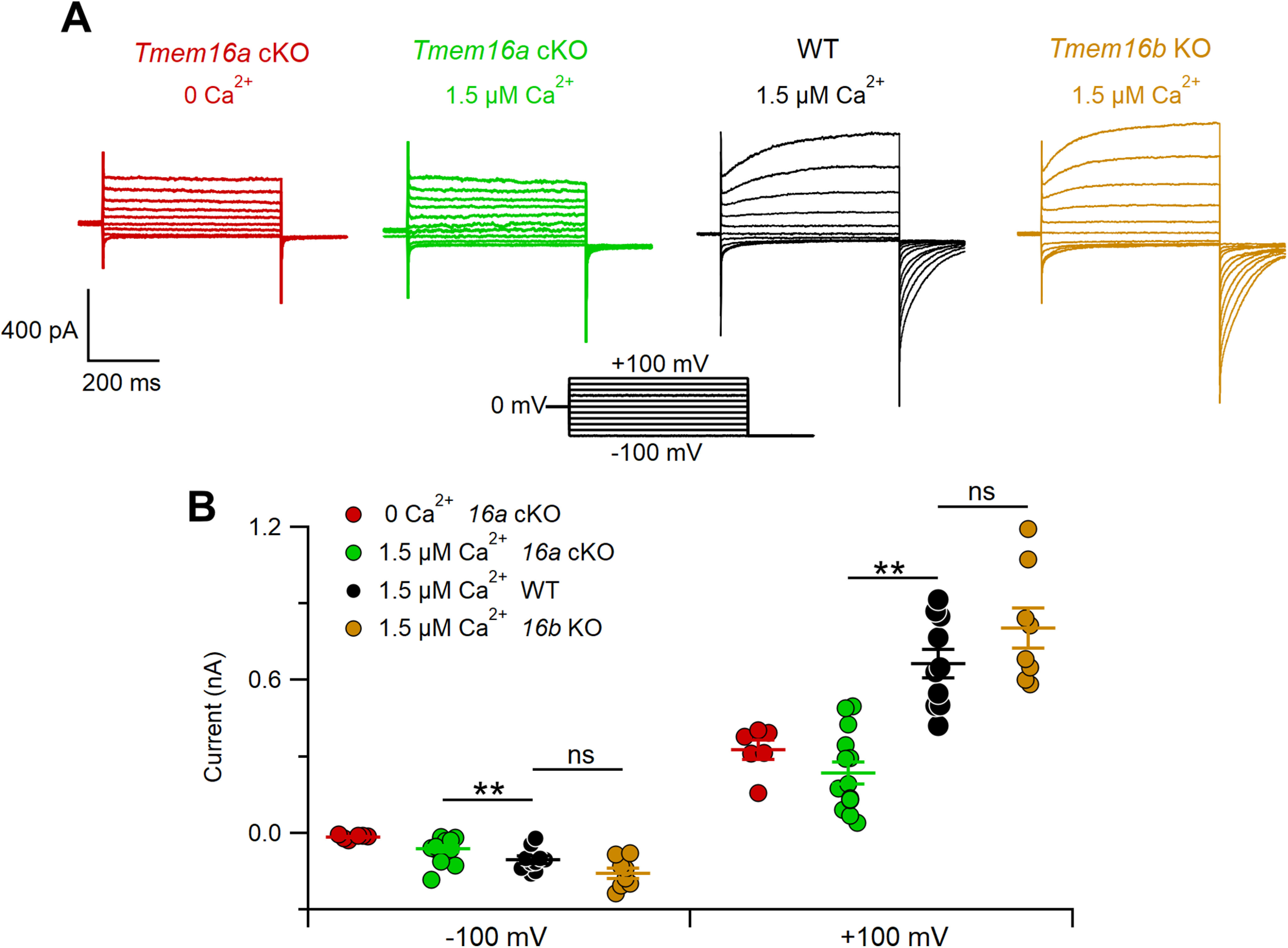
Ca^2+^-activated chloride currents in VSNs. ***A***, Representative whole-cell recordings obtained with an intracellular solution containing 0 or 1.5 μm Ca^2+^ from VSNs from WT, *Tmem16a* cKO, or *Tmem16b* KO mice, as indicated. The voltage protocol is in the center of the figure. ***B***, Scatter dot plot with average ± SEM of steady-state current amplitudes measured at −100 or +100 mV with intracellular pipette solution containing nominally 0 Ca^2+^ from *Tmem16a* cKO (red, *n* = 6), or 1.5 μm free Ca^2+^ from *Tmem16a* cKO (green, *n* = 14), WT (black, *n* = 10), and *Tmem16b* KO (brown, *n* = 8) mice (**p < 0.01; Dunn–Hollander–Wolfe test after Kruskal–Wallis analysis at –100 and +100 mV), ns: not significant.

To determine the respective contributions of TMEM16A and TMEM16B to Ca^2+^-activated chloride currents in VSNs, we used intracellular solutions with nominally 0 Ca^2+^ or with 1.5 μm free Ca^2+^ and obtained whole-cell voltage-clamp recordings from the soma of neurons in acute VNO slices from WT, *Tmem16a* cKO, and *Tmem16b* KO mice (Fig. 2). To avoid contributions from K^+^ currents (Ca^2+^-activated or voltage-activated), the pipette solution contained Cs^+^ instead of K^+^. Figure 2*A* shows representative VSN recordings (holding potential V_hold_ = 0 mV) from each mouse line at voltage steps between −100 and +100 mV, followed by a step to −100 mV. In agreement with previous results (Amjad et al., 2015), we confirmed that VSNs from *Tmem16a* cKO mice lacked a significant Ca^2+^-activated chloride current in the presence of 1.5 μm Ca^2+^. Indeed, the average currents measured at steady state in 0 Ca^2+^ (−15 ± 3 pA at −100 mV and 326 ± 38 pA at +100 mV, *n* = 6) or in 1.5 μm Ca^2+^ (−62 ± 13 pA at −100 mV and 236 ± 41 pA at +100 mV, *n* = 14) were not significantly different (Fig. 2*B*). Furthermore, VSN currents activated by voltage steps in *Tmem16a* cKO mice did not show any time dependence (red and green traces), whereas WT currents in 1.5 μm Ca^2+^ (black traces) showed the typical kinetics characterized by an instantaneous current followed by an outward or inward relaxation at the most depolarizing or hyperpolarizing voltage steps, respectively. Recordings from VSNs of *Tmem16b* KO mice (brown traces; −157 ± 20 pA at −100 mV and 805 ± 79 pA at +100 mV, *n* = 8) revealed Ca^2+^-activated chloride currents similar to WT mice (−105 ± 14 pA at −100 mV and 666 ± 55 pA at +100 mV, *n* = 10), further corroborating previous findings that TMEM16A largely contributes to Ca^2+^-activated chloride currents in VSNs (Fig. 2*B*).

### Membrane properties and voltage-gated currents of VSNs from KO mice are not altered

To test whether *Tmem16a* or *Tmem16b* deletion changes VSN passive membrane properties and/or voltage-gated currents, we performed whole-cell recordings in the presence of K^+^ in the patch pipette.

**Figure 3.**
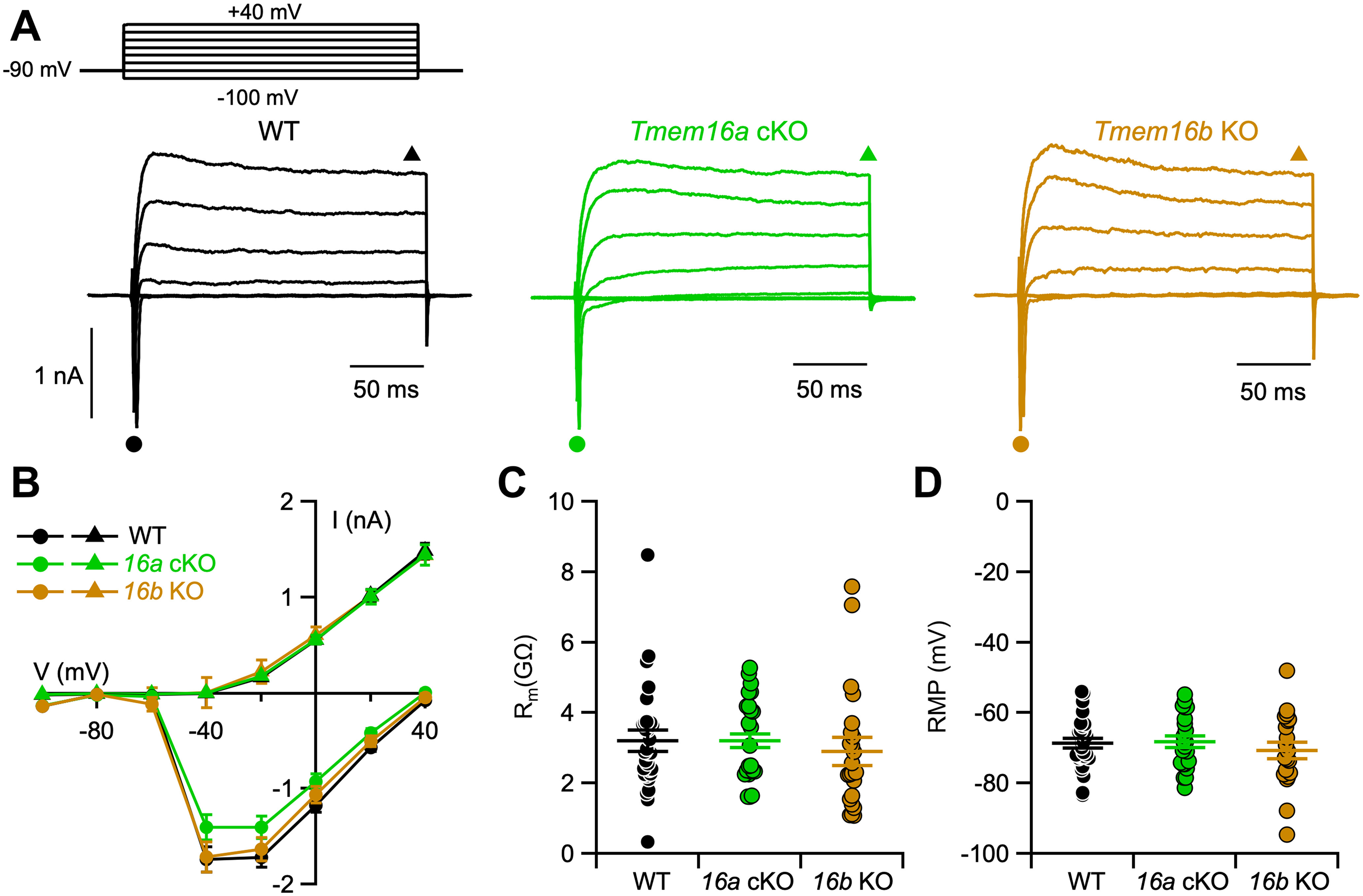
*Tmem16a* or *Tmem16b* deletion does not affect voltage-gated currents and membrane properties of VSNs. ***A***, Representative whole-cell recordings of VSNs obtained from the indicated mouse lines. The holding potential was −90 mV and voltage steps from −110 to +30 mV with 20-mV increment were applied. ***B***, Average ± SEM of the IV relationships of inward currents (circles) and steady-state outward currents (triangles) from WT (black, *n* = 31), *Tmem16a* cKO (green, *n* = 23), or *Tmem16b* KO (brown, *n* = 23) VSNs. Values were taken at the negative peak and at the end of voltage step as indicated by the symbols in ***A***. ***C***, Scatter dot plot with average ± SEM of the membrane resistance (R_m_) recorded in voltage-clamp mode (WT, *n* = 31; *Tmem16a* cKO, *n* = 23; *Tmem16b* KO, *n* = 23. Kruskal–Wallis analysis *p* = 0.21). ***D***, Scatter dot plot with average ± SEM of resting membrane potential (RMP) recorded in current-clamp configuration (WT, *n* = 28; *Tmem16a* cKO, *n* = 22; *Tmem16b* KO, *n* = 20. Kruskal–Wallis analysis *p* = 0.68).

Representative recordings of voltage-activated inward and outward currents from each mouse line are shown in Figure 3*A*. For I-V relations, we plotted the minimum values of inward currents during the first 20 ms after each voltage pulse (Fig. 3*B*, circles) and the outward current value at the end of each voltage pulse (Fig. 3*B*, triangles). Figure 3*B* shows that I-V relations of voltage-gated currents were similar in VSNs from WT, *Tmem16a* cKO, and *Tmem16b* KO mice.

Membrane input resistance was evaluated in voltage-clamp mode with a negative pulse of 20 mV from a holding potential of −90 mV, as in these conditions voltage-gated currents are not activated (Dibattista et al., 2008). The average membrane input resistances of VSNs from WT, *Tmem16a* cKO, and *Tmem16b* KO mice were 3.2 ± 0.3 GΩ (*n* = 31), 3.2 ± 0.2 GΩ (*n* = 23), and 2.9 ± 0.4 GΩ (*n* = 23), respectively (Fig. 3*C*). These values were not statistically different, indicating that membrane input resistance is not altered by either the lack of TMEM16A or TMEM16B.

The resting membrane potential was measured in current-clamp mode (*I*_inject_ = 0 pA) and was not statistically different between genotypes (Fig. 3*D*), with values of −69 ± 1 mV (*n* = 28) for WT, −68 ± 2 mV (*n* = 22) for *Tmem16a* cKO, and −71 ± 2 mV (*n* = 20) for *Tmem16b* KO VSNs.

These results indicate that the Ca^2+^-activated chloride channels TMEM16A and TMEM16B do not contribute to passive membrane properties of mouse VSNs and do not alter major voltage-gated inward and outward currents in the range from −100 up to +40 mV. As TMEM16A can also be activated by high positive voltages in absence of intracellular Ca^2+^ (Xiao et al., 2011; Segura-Covarrubias et al., 2020), these results show that TMEM16A does not alter passive properties at membrane potentials up to +40 mV.

### Spontaneous firing pattern is altered in VSNs from *Tmem16a* cKO

It is well known that VSNs display spontaneous firing activity in the absence of stimuli (Holy et al., 2000; Arnson and Holy, 2011) and that changes in sensory transduction components may modify this activity, as for example VSNs from *Trpc2* KO mice showed reduced spontaneous firing (Arnson and Holy, 2011). Moreover, experiments in the main olfactory epithelium have shown that deletion of TMEM16B, which is highly expressed in the cilia of olfactory sensory neurons (OSNs), caused a reduction of spontaneous firing activity in those neurons (Pietra et al., 2016). Based on these findings, we hypothesized that TMEM16A and TMEM16B might play a role in the spontaneous VSN firing.

**Figure 4.**
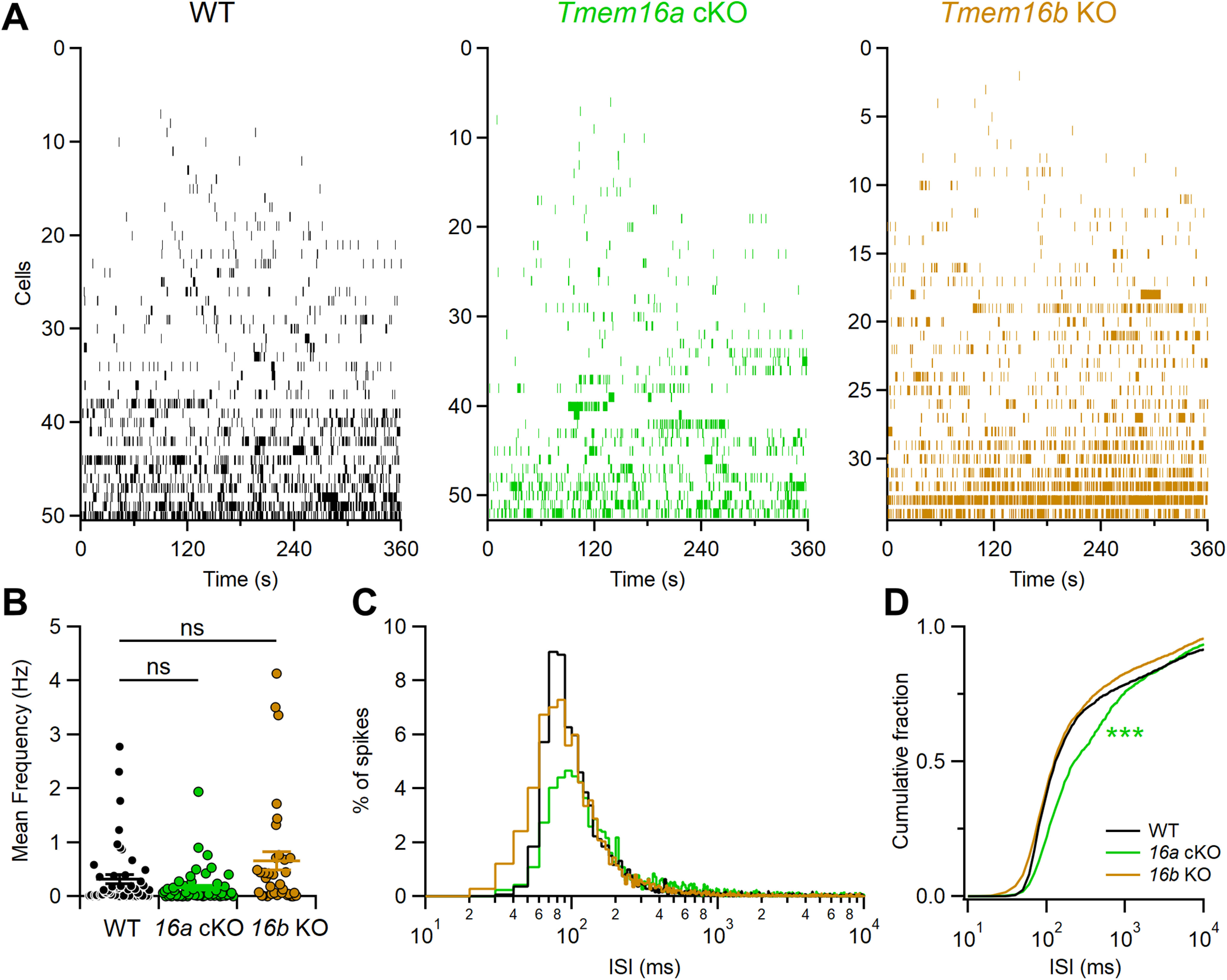
*Tmem16a* deletion modifies VSNs spontaneous firing activity. ***A***, Raster plots of recordings of the spontaneous activity of VSNs from WT (black, *n* = 50), *Tmem16a* cKO (green, *n* = 52), or *Tmem16b* KO (brown, *n* = 34) mice. Each row shows spike activity from a different VSN. ***B***, Mean frequency of spontaneous activity from the cells shown in ***A***. ***C***, ISI distributions of spontaneous firing from a subset of cells with mean frequency >0.1 Hz (bin = 10 ms). Values were normalized to the area under each curve to show the spike percentages for WT (black, *n* = 25), *Tmem16a* cKO (green, *n* = 18), or *Tmem16b* KO (brown, *n* = 20) mice. ***D***, Cumulative fraction of ISI distributions (***p < 0.001, Kolmogorov–Smirnov test).

By performing extracellular loose patch recordings from individual neurons in VNO slices from WT and mutant mice, we found that VSNs have a highly variable spontaneous activity among neurons of the same genotype with frequencies varying from 0 Hz (no spontaneous activity) to ∼4 Hz (Fig. 4*A*). The viability of each neuron was tested by depolarization with a high K^+^ solution to verify the neurons firing capability, and only VSNs responding to high K^+^ were included in the analysis. VSNs that did not show spontaneous activity were: six out of 50 for WT, five out of 52 for *Tmem16a* cKO, and one out of 34 for *Tmem16b* KO. Thus, at least 88% VSNs from each genotype showed spontaneous activity.

Although the average spontaneous frequency was not significantly different among the three genotypes (0.32 ± 0.08 Hz, *n* = 50 for WT; 0.16 ± 0.04 Hz, *n* = 52 for *Tmem16a* cKO, and 0.65 ± 0.18 Hz, *n* = 34 for *Tmem16b* KO; Fig. 4*B*), we measured differences in firing patterns. To analyze activity patterns, we calculated ISI distributions from neurons with a mean frequency >0.1 Hz and plotted the distributions for each mouse line (Fig. 4*C*). To avoid overrepresentation of neurons with higher versus those with lower spontaneous activity, we normalized the ISI distribution by dividing the number of spikes for each bin by the total number of spikes for each single VSN before adding it to create the ISI distribution for each genotype. Moreover, to compare data from the three genotypes, data were normalized to the area under the curve (Fig. 4*C*). Corresponding cumulative fraction plots are shown in Figure 4*D*. We found that ISI distributions of spontaneous firing were statistically different (*p* < 0.01) between WT and *Tmem16a* cKO VSNs, while they did not differ between WT and *Tmem16b* KO VSNs. Indeed, in WT and *Tmem16b* KO VSNs, ∼80% of ISIs range between 50 and 200 ms (black and brown traces, respectively). By contrast, the ISI distribution for *Tmem16a* cKO neurons is broader showing only ∼50% of activity in the 50- to 200-ms ISI range (green traces). These results show that short ISIs were reduced in *Tmem16a* cKO compared with WT neurons (Fig. 4*C*,*D*), indicating that TMEM16A, but not TMEM16B, exerts firing pattern control during spontaneous activity of VSNs.

### Deletion of TMEM16A and resulting changes in spontaneous firing do not modify axonal targeting to AOB glomeruli

**Figure 5.**
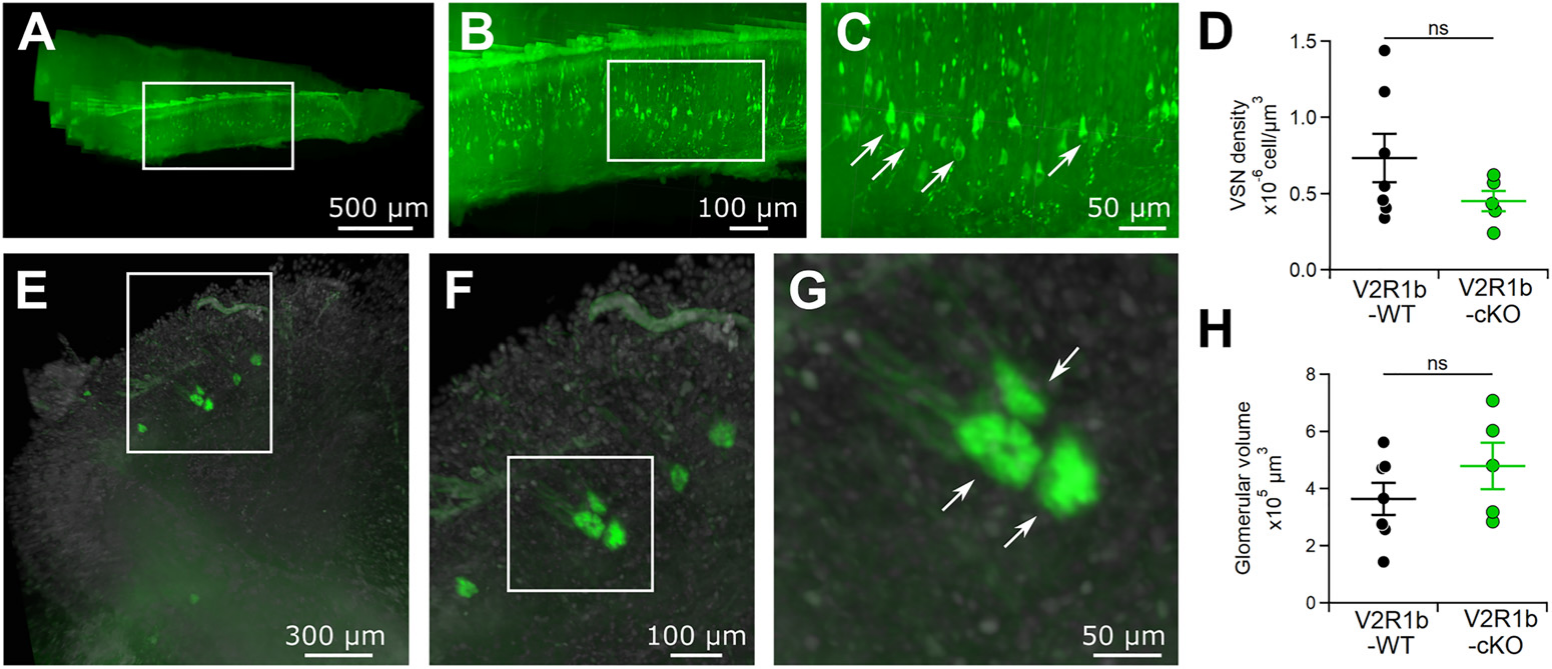
*Tmem16a* cKO effect on VSN density and glomerular volume. ***A***–***C***, top, Representative 3D projection image of a VNE from a V2R1b-WT mouse. Zoom-in indicated by white rectangles. Arrows indicate example fluorescently labeled V2R1b neurons. ***D***, Scatter dot plot (average ± SEM) of VSN density in V2R1b-WT and V2R1b-*Tmem16a* cKO mice (Student’s *t* test, *p* = 0.1876). ***E***–***G***, top, Representative 3D projection image of AOB from V2R1b-WT mouse. Zoom-in indicated by white rectangles. Arrows indicate exemplary fluorescently labeled V2R1b glomerular structures. ***H***, Scatter dot plot with average ± SEM of glomerular volume in V2R1b-WT and V2R1b-*Tmem16a* cKO mice (Student’s *t* test, *p* = 0.2573).

In the main olfactory epithelium, spontaneous activity of OSNs is essential for both formation and maintenance of the glomerular map in the OB (Yu et al., 2004; Lodovichi and Belluscio, 2012; Lorenzon et al., 2015). Furthermore, deletion of TMEM16B, the Ca^2+^-activated chloride channel in OSNs, altered spiking and affected the glomerular formation of I7-expressing OSNs (Pietra et al., 2016). While the relation between spiking pattern and glomerular formation has been investigated in the OB, similar studies in the AOB were missing. To investigate whether spontaneous firing pattern alteration, resulting from TMEM16A deletion, affects VSN density or glomeruli formation in the AOB, we crossed *Tmem16a* cKO with V2R1b-GFP (Del Punta et al., 2002) mice to generate a mouse line in which V2R1b expressing VSNs were fluorescently labeled, but lack TMEM16A (V2R1b-GFP-TMEM16A-cKO). V2R1b-GFP-TMEM16A cKO mice were homozygous for V2R1b-GFP and *Tmem16a*-Flox genes, and heterozygous for OMP/Cre, while control animals were homozygous for V2R1b-GFP, *Tmem16a*-Flox and OMP genes, lacking the expression of Cre recombinase. We performed VNO and forebrain tissue clearing to quantify and compare (1) the number of fluorescently labeled neurons per VNO, and (2) the V2R1b-specific axonal glomerular architecture in the AOB, between TMEM16A-deficient V2R1b-GFP mice and corresponding controls (Fig. 5). Figure 5*A–C* shows an example of a cleared VNE observed with a light sheet microscope and the corresponding 3D reconstruction (Movie 1). Single VSNs were readily observed, and cellular density was calculated (Fig. 5*D*). We found no significant statistical difference in VSN density between V2R1b-GFP-WT and V2R1b-GFP-TMEM16A-cKO mice (Fig. 5*D*, *n* = 5–7). These results indicate that deletion of TMEM16A does not affect the number of V2R1b-expressing VSNs.

Next, we evaluated the glomerular architecture in the AOB. We identified several glomeruli innervated by V2R1b axons, confirming previous reports (Del Punta et al., 2002). Given the variability in glomerular number between animals from the same mouse line, ranging between 2 and 10 glomeruli per AOB (Del Punta et al., 2002), we decided to evaluate whether the total volume of AOB glomeruli was affected by TMEM16A deletion. Figure 5*E–G* shows a representative 3D reconstruction of the AOB with glomeruli formed by V2R1b-GFP axons (Movie 2). Single glomeruli were discernable (Fig. 5*G*, white arrows). A comparison of GFP-positive glomerular volume between V2R1b-WT and V2R1b-cKO mice show no significant statistical difference (Fig. 5*H*, *n* = 5–6). These results indicate that deletion of TMEM16A protein in VSNs and resulting alterations in firing do not affect general glomerular development in the AOB.

Movie 1. Light-sheet fluorescence microscopy of cleared VNOs. Examples show intact transparent organs from wild type control (V2R1b-GFP - TMEM16Afl/fl - OMP/OMP; left) and TMEM16A knockout (V2R1bGFP - TMEM16Afl/fl - OMP/Cre; right) animals. Three-dimensional rendering displays the VNOs rotating around their longitudinal axes. 10.1523/ENEURO.0179-21.2021.video.1

Movie 2. Light-sheet fluorescence imaging of cleared AOBs. Examples show intact transparent olfactory bulb samples from wild type control (V2R1b-GFP - TMEM16Afl/fl - OMP/OMP; left) and TMEM16A knockout (V2R1bGFP - TMEM16Afl/fl - OMP/Cre; right) mice. Nuclei are stained with DRAQ5 (grey scale). Green fluorescence of V2R1b-positive vomeronasal sensory neuron axons becomes apparent upon fiber coalescence and convergence in AOB glomeruli. 10.1523/ENEURO.0179-21.2021.video.2

### Response to sensory stimulation is altered in VSNs from KO mice

**Figure 6.**
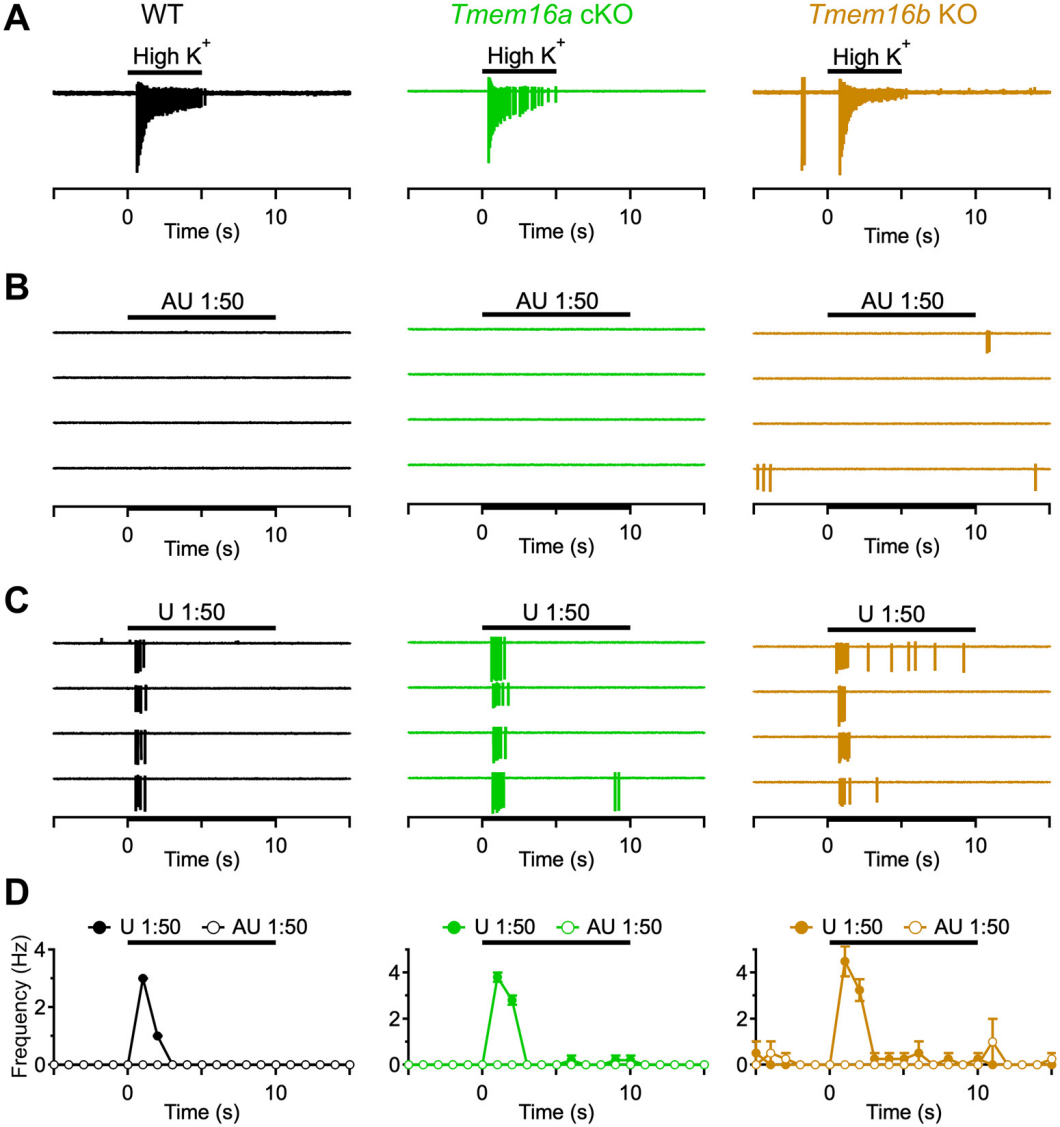
Evoked firing activity. ***A****–****C***, Representative loose-patch recordings of a VSN from each indicated mouse line stimulated with high K^+^ (***A***), diluted artificial urine as control (***B***), or diluted urine (***C***). Black bars indicate the time of stimulus presentation. ***D***, Average firing activity (1-s bin width) for the responses to diluted artificial urine (empty circles) or urine (filled circles) from ***B***, ***C***. Error bars represent SEM.

Next, we asked whether TMEM16A or TMEM16B deletion affects VSN responses to sensory stimulation. Thus, we stimulated VSNs with diluted mouse urine, a natural source of pheromones that has been frequently used to activate VSNs (Mohrhardt et al., 2018). While recording in loose-patch configuration, we stimulated VSNs with diluted urine and recorded the evoked firing activity. Stimulus application was repeated at least three times to avoid the detection of false-positive responses in spontaneously active neurons (Fig. 5). The viability of each VSN was tested by using a short depolarizing high K^+^ solution stimulus (Fig. 6*A*), where unresponsive neurons were discarded. Furthermore, as urine also contains various ions including K^+^ that could depolarize neurons (see Materials and Methods), we stimulated VSNs with diluted artificial urine (Fig. 6*B*) Neurons responding to the artificial urine control stimulus were excluded from the analysis. Finally, as VSNs display spike frequency adaptation to repetitive stimulation (Wong et al., 2018), we selected an interstimulus interval between urine application of 4 min to avoid adaptation.

**Figure 7.**
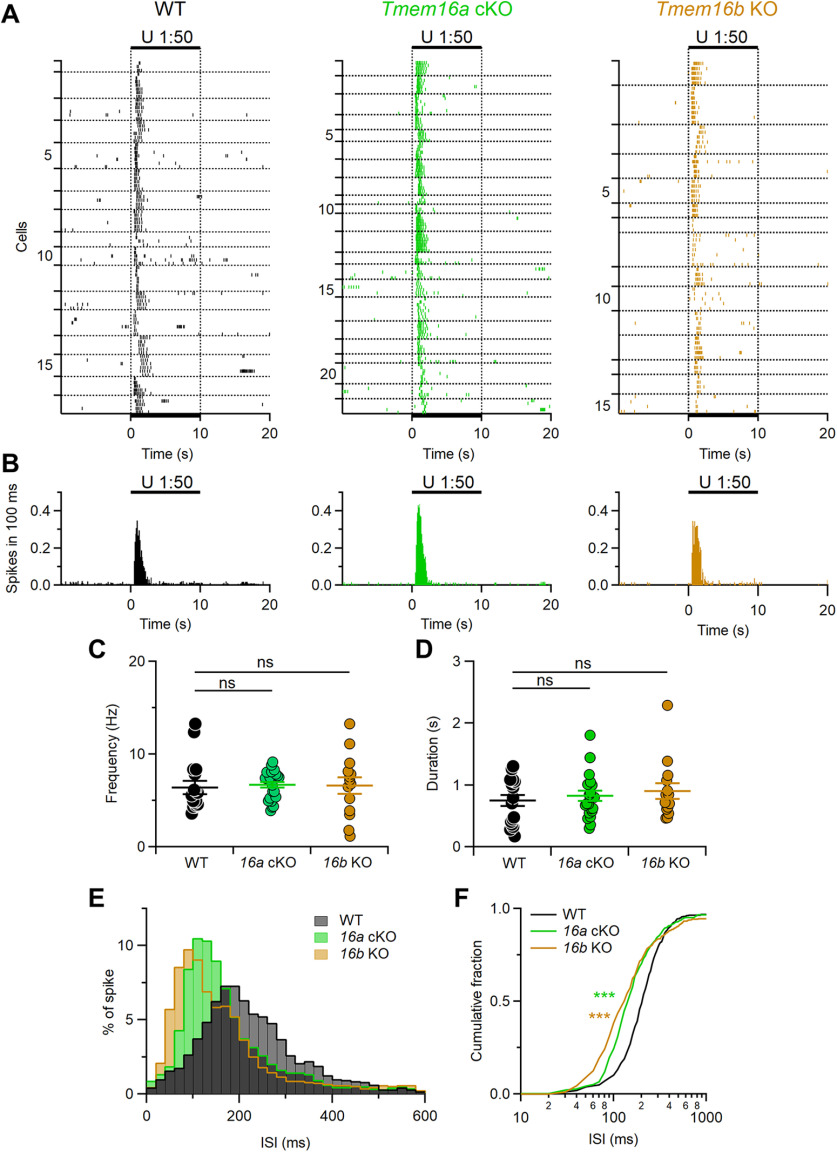
Patterns of evoked firing activity are modulated by TMEM16A and TMEM16B. ***A***, Raster plots showing the responses of VSNs from each indicated mouse line to diluted urine. Black bars indicate the time of stimulus presentation (10 s). Every neuron was tested at least three times. Horizontal dotted lines delimit multiple recordings from each VSNs. Numbers on the left indicate the neuron number. ***B***, Histograms of the number of spikes from neurons in ***A*** (100-ms bin width). Each neuron was averaged to equally contribute to spikes count. ***C***, ***D***, Scatter dot plots with average ± SEM of the mean frequency (***C***, Kruskal–Wallis analysis *p* = 0.37) and duration (***D***, Kruskal–Wallis analysis *p* = 0.79) of the responses to urine of VSNs from WT (black, *n* = 17), *Tmem16a* cKO (green, *n* = 22), or *Tmem16b* KO (brown, *n* = 15) mice. ***E***, Normalized ISI distributions of firing activity (20-ms bin width). ***F***, Cumulative fraction of the ISI distributions shown in ***D*** (***p < 0.001, Kolmogorov–Smirnov test).

Figure 6 shows representative responses of single VSNs from WT or mutant mice. A 10-s stimulus of diluted urine was applied four times and, each time, clearly elicited an increase in firing frequency (Fig. 6*C*), whereas application of diluted artificial urine did not alter spiking activity (Fig. 6*B*). The plot of average firing frequency (1-s-bin width) shows a clear frequency increase during urine application in VSNs from either genotype (Fig. 6*D*).

Figure 7 shows reliable responses to repeated urine stimulation in 17 out of 30 WT, 22 out of 30 *Tmem16a* cKO, and 15 out of 34 *Tmem16b* KO VSNs, indicating that >45% VSNs from each genotype can respond to urine (Fig. 7*A*,*B*).

Although the mean firing frequency and response duration to urine were not significantly different among genotypes (Fig. 7*C*,*D*) a more detailed analysis showed that patterns of firing responses were altered by deletion of TMEM16A or TMEM16B (Fig. 7*E*,*F*). Indeed, ISI distributions and cumulative fractions of mutant mice were different from those of WT. ISI distribution for WT showed a peak ∼200 ms, while in the ISI distribution from *Tmem16a* cKO and *Tmem16b* KO the peak was shifted to a shorter ISI of ∼100 ms. These results indicate that both TMEM16A and TMEM16B modulate firing activity during signal transduction in mouse VSNs.

## Discussion

In this study, we investigated the individual roles of TMEM16A and TMEM16B in regulating the physiological activity of VNO neurons. We found that TMEM16A, but not TMEM16B, modulates spontaneous spike patterns by increasing activity, while both channels shape pheromone-mediated spike activity by increasing ISIs.

### Expression of TMEM16A and TMEM16B in olfactory systems

While the presence of Ca^2+^-activated chloride currents in neurons of the main olfactory system is known since the early 1990s (Kleene and Gesteland, 1981; Kleene, 1993; Kurahashi and Yau, 1993; Lowe and Gold, 1993; Boccaccio and Menini, 2007; Pifferi et al., 2012; Dibattista et al., 2017), the first recordings of Ca^2+^-activated currents from VSNs have been obtained more recently (Yang and Delay, 2010; Kim et al., 2011; Dibattista et al., 2012; Amjad et al., 2015).

Ca^2+^-activated chloride channels encoded by TMEM16A and TMEM16B are characterized by functional differences. These include slower activation kinetics and higher Ca^2+^ sensitivity for TMEM16A compared with TMEM16B. The complexity is further increased by the existence of several spice variants with different properties (Ferrera et al., 2009; Huang et al., 2012; Ponissery Saidu et al., 2013; Pedemonte and Galietta, 2014).

In the main olfactory epithelium, TMEM16A and TMEM16B are expressed in different cell types: TMEM16B is expressed in the dendritic ends and cilia of OSNs (Stephan et al., 2009; Sagheddu et al., 2010; Billig et al., 2011), whereas TMEM16A is present in apical membranes and microvilli of a subpopulation of supporting cells (Henriques et al., 2019; Maurya and Menini, 2014). In the vomeronasal epithelium, however, both TMEM16A and TMEM16B are found in VSNs (Billig et al., 2011; Dibattista et al., 2012; Amjad et al., 2015; Münch et al., 2018), where they co-express in microvilli (Dibattista et al., 2012; Münch et al., 2018). Here, we confirmed previous results that TMEM16A is present only in mature VSNs, but not in VNO supporting cells (Amjad et al., 2015; Münch et al., 2018). By taking advantage of *mCherry Tmem16b* KO mice (Zhang et al., 2017), we showed that TMEM16B is also selectively expressed in mature VSNs. Thus, vomeronasal TMEM16A and TMEM16B are exclusively expressed in mature VSNs. The relative expression level of the two channels has been evaluated in the transcriptomic study of Ibarra-Soria et al. (2014). They found high expression for both transcripts, with *Tmem16a* levels about twice that of *Tmem16b*, without significant difference between males and females (Ibarra-Soria et al., 2014; see their Dataset S1 and Dataset S3).

In OSNs, deletion of TMEM16B completely abolished Ca^2+^-activated chloride currents measured in whole-cell recordings (Billig et al., 2011; Pietra et al., 2016). In VSNs, the deletion of TMEM16A also completely abolished the current activated by 1.5 μm Ca^2+^ and measured with voltage steps up to +100 mV. As VSNs also express TMEM16B we expected to measure substantial residual current in the absence of TMEM16A. Indeed, although TMEM16B is less sensitive to Ca^2+^ than TMEM16A, it should be activated by 1.5 μm Ca^2+^ at +100 mV, as an EC_50_ of ∼2 μm has been reported at high voltages (Cenedese et al., 2012; Adomaviciene et al., 2013). We also attempted to increase intracellular Ca^2+^ to 13 and 100 μm, respectively, but VSNs in slices became leaky with these solutions. However, previous experiments in isolated VSNs successfully increased intracellular Ca^2+^ up to 2 mm without finding any measurable Ca^2+^-activated chloride current in VSNs from *Tmem16a* cKO mice (Amjad et al., 2015). Yet, as the average whole-cell leak current at +100 mV was ∼300 pA, we cannot exclude that we missed a small current activated by TMEM16B. Lack of current in *Tmem16a* cKO mice was also measured by Münch et al. (2018) who suggested the possibility that Ca^2+^ concentration in the microvilli does not reach the concentration necessary to activate TMEM16B. We found that deletion of TMEM16B did not significantly reduce Ca^2+^-activated chloride currents in VSNs confirming that most current results from TMEM16A activation. Our result thus agrees with recently published findings (Münch et al., 2018). By contrast, our data contradict a previous report showing a lack of Ca^2+^-activated chloride currents in VSNs from *Tmem16b* KO mice (Billig et al., 2011).

It is of interest to note that TMEM16A and TMEM16B are co-expressed in pinealocytes where they can form both homomeric and heteromeric channels (Yamamura, 2018), raising the possibility that VSNs also express heteromeric channels.

### Spontaneous and stimulus-evoked spike activity

In both OSNs and VSNs, Ca^2+^-activated chloride channels are expected to play a role in the response to odorants or pheromones, as they are located close to transduction sites, i.e., in OSN cilia and VSN microvilli. This subcellular distribution is well suited to sense high Ca^2+^ concentrations produced in microdomains by transduction cascades downstream odorant or vomeronasal receptor activation (Tirindelli et al., 2009; Pifferi et al., 2010). Indeed, although the transduction cascade components in the two olfactory systems are very different, transduction currents produce an increase in intracellular Ca^2+^ in both cases. The resulting membrane depolarization triggers generation of action potentials that are conducted along OSN or VSN axons to the main or AOB, respectively. To understand the physiological role of TMEM16 channels in peripheral sensory neurons of olfactory systems, it is critical to note that both OSNs and VSNs maintain high internal chloride concentrations (Reuter et al., 1998; Kaneko et al., 2004; Kim et al., 2015; Untiet et al., 2016). Therefore, Ca^2+^-activated chloride channels are well suited to play an excitatory transduction role by amplifying the depolarizing cation current through chloride efflux. In addition, these channels might also play a stabilizing role by setting the membrane potential close to the chloride equilibrium potential, preventing excessive depolarization.

In OSNs, extracellular loose patch recordings of spontaneous firing activity showed that TMEM16B increased spontaneous firing, as TMEM16B-deficient OSNs exhibited a reduction in instantaneous frequency (inverse of ISI) of spontaneous activity (Pietra et al., 2016). This likely results from random activation of signal transduction proteins that produces a Ca^2+^ increase sufficient to open ciliary TMEM16B channels that contribute small depolarizations and firing activity. As VSNs also display a spontaneous activity that is dependent on signal transduction cascade components (Arnson and Holy, 2011), we hypothesized that TMEM16 channels could also affect spontaneous VSN firing. Indeed, deletion of TMEM16A, but not of TMEM16B, caused differences in ISI distribution: the percentage of ISIs between 50 and 200 ms was reduced to 50%, compared with 80% in WT VSNs. The decrease in spontaneous activity after TMEM16A deletion indicates that TMEM16A favors spike generation, probably by mediating a chloride efflux that further depolarizes the membrane after spontaneous activation of signal transduction components, similarly to TMEM16B in OSNs. As TMEM16A has a higher Ca^2+^ sensitivity than TMEM16B, a possible explanation for the lack of contribution of TMEM16B in VSNs is that the spontaneous activation of the signal transduction cascade leads to intracellular Ca^2+^ increase, which is insufficient to activate TMEM16B. Moreover, *Tmem16b* transcript expression level has been reported to be half of that of *Tmem16a* in VSNs (Ibarra-Soria et al., 2014) indicating that TMEM16B, in addition to being less sensitive to Ca^2+^, may also have a lower expression than TMEM16A.

In the main olfactory system, TMEM16B is also involved in correct OSN glomerular targeting, as its deletion caused the formation of supernumerary glomeruli for neurons expressing the I7 odorant receptor (Pietra et al., 2016). In the vomeronasal system, deletion of TMEM16A did not produce modifications of glomerular targeting for VSNs expressing the V2R1b receptor, although we cannot exclude the possibility that this deletion may affect glomerular targeting of VSNs expressing other receptors.

In OSNs, TMEM16B has been shown to have a dual role in the control of firing, by increasing basal spiking but reducing stimulus-induced firing activity. In VSNs, we found that TMEM16A also plays a similar dual role. Indeed, individual loss of TMEM16A (or TMEM16B) strongly affected urine-evoked firing patterns by shifting the peak in the ISI distribution to ∼100 ms from the value of 200 ms in WT. Thus, both TMEM16A and TMEM16B channels play a role in regulating VSN firing activity evoked by natural stimuli by doubling ISI. Stimulation of VSNs with diluted urine can forcefully activate the signal transduction cascade generating a stronger depolarization and higher cytosolic Ca^2+^ increase compared with spontaneous activation; under these conditions, TMEM16A and/or TMEM16B could first mediate a chloride efflux that amplifies the depolarization followed by a chloride influx, when the membrane potential becomes more positive than the chloride equilibrium potential, leading to membrane repolarization and increase in the ISI. Indeed, the internal chloride concentration in dendritic knobs of VSNs at rest has been estimated to be 42 mm (Untiet et al., 2016). In our experimental conditions, this would produce an equilibrium potential for chloride near −30 mV. Consequently, opening of Ca^2+^-activated chloride channels would lead to depolarization at lower membrane potential and to repolarization at increased membrane potential.

Another study used *Tmem16a*/*Tmem16b* double KO mice (Münch et al., 2018) and reported a loss of spontaneous spiking and drastically diminished stimulus evoked spiking in VSNs indicating a relevant physiological role of these channels. Recordings and/or data analysis were somehow different from ours as they reported that only ∼30% of VSNs had spontaneous activity while we measured spontaneous activity in almost 90% of VSNs from WT mice, in agreement with previous reports (Holy et al., 2000; Nodari et al., 2008; Arnson and Holy, 2011; Wong et al., 2018). This difference could stem from either sample viability, as depolarization controls were not reported, or different recording conditions. A more detailed study of firing activity should be performed in VSNs from the double KO model to better elucidate firing patterns. The same study performed a behavioral test showing that male-male territorial aggression, an innate VNO-dependent behavior in rodents, remained unaltered in double KO *Tmem16a*/*Tmem16b* mice.

It is important to note that our criteria for selecting neurons responding to urine are more stringent with respect to those of other studies (Kim et al., 2011; Münch et al., 2018) and could have excluded some VSNs with very high spontaneous firing frequencies. We argue that our method, similar to that used by Arnson and Holy (2011), is suitable to limit the analysis to a subset of clearly viable neurons.

In summary, our data provide evidence that both TMEM16A and TMEM16B modulate the pheromone-mediated spike activity of VSNs and provide the foundation for future work investigating the precise physiological role of the Ca^2+^-activated chloride currents in VSNs.

## Synthesis

Reviewing Editor: Christina Zelano, Northwestern University Decisions are customarily a result of the Reviewing Editor and the peer reviewers coming together and discussing their recommendations until a consensus is reached. When revisions are invited, a fact-based synthesis statement explaining their decision and outlining what is needed to prepare a revision will be listed below. The following reviewer(s) agreed to reveal their identity: Stephan Frings, Haiqing Zhao.

Both reviewers agreed that this is an interesting study that is a significant advancement in the field, as it provides the first high-quality data on the question of how pheromone-induced signals are modulated by calcium-dependent chloride currents ín pheromone-sensitive neurons. The reviewers had some important comments that can be addressed by adding some comments and explanations to the text. They also asked for some clarifications of the methods, which need to be added to the manuscript. Please see below the original comments from the reviewers:

Reviewer 1

The authors present evidence for a modulatory role of calcium-activated chloride channels in signal generation by vomeronasal neurons in mice. The interest of this story lies in the fact that the output signals of sensory neurons are often finely-tuned by calcium-dependent ion channels, and there is growing evidence that TMEM16-type calcium-activated chloride channels serve such a purpose. For mouse VNO neurons, the authors show that the channels basically double interspike intervals, thus putting the brake on afferent signaling. Following a small series of papers on TMEM16 channels in VNO neurons, the present manuscript provides the hitherto most reliable data and is suited to provide a first plausible working hypothesis. While several aspects of this report require additional attention, the results are important for the fields of neuromodulation and sensory physiology.

Points that need to be addressed in revision:

1. The authors suggest that two different TMEM16 ion channels operate in VNO neurons, a notion that is well founded by previous studies. According to literature, one of the main functional differences between the two is a 10-fold difference in calcium sensitivity, with concentrations for half-maximal activation of about 0.2 μM in TMEM16A and near 2 μM in TMEM16B. In their whole-cell experiments, the authors use a calculated (not measured) calcium concentration of 1.5 μM, which is expected to saturate TMEM16A, but cause little activation of TMEM16B. This approach is problematic as the real calcium concentration may differ substantially from the calculated one (3.029 mM CaCl + 10 mM HEDTA, pH 7.2), an uncertainty that all researchers face when working with cell calcium. In the present context, this point is of particular relevance because the “1.5 μM calcium” solution may indeed selectively activate TMEM16A, but not TMEM16B. This point must be made transparent for the reader. If, for example, whole-cell traces in Fig. 2 are similar in Tmem16b-ko and wild-type neurons, this does not indicate that TMEM16B is not expressed, but it may simply indicate that the calcium concentration within the pipette solution was not sufficiently high to activate TMEM16B. In fact, if the authors were interested in looking for TMEM16B currents, they should have used a different calcium concentration (10 μM) in an additional, separate set of recordings. That would have been wise, as all the interesting effects of the chloride channels reported later in the manuscript could be explained with two channel types of different calcium sensitivities.

2. The control recordings to determine possible changes of input resistance and resting membane potential at zero calcium are well done but lack, to my understanding, one aspect. TMEM16A was reported to open at positive voltages even in calcium-free solution. The voltage-clamp recordings in Fig. 3 show that this does not alter passive properties up to +40 mV, a voltage range where AP dynamics can conceivably be modulated by chloride currents. The authors demonstrate that this is not the case and may consider attracting the reader´s attention to this point.

3. To check whether glomerular targeting in Tmem16a-ko mice was disturbed, the authors crossed two mouse lines to obtain V2R1b-GFP-TMEM16A-cKO animals. To assess the validity of these experiments, readers need to know which generation of cross-breeding was used. In particular, was this line heterozygous with respect to Tmem16a so that one intact Tmem16a allele was present? In that case, the lack of effect on OE/OB wiring could be explained by the remaining allele.

4. A strength of this study was that the authors selected viable, responsive VNO neurons for examination, discarding all cells that were either unresponsive or did not fit control criteria (like the lack of response to artificial urine). The success of the study clearly justifies that strategy. The authors may consider adding to the Discussion a statement on how they assess the possibility that such a strict selection procedure can introduce a bias to the outcome of the study.

5. The authors shy away from specifying a distinct working hypothesis on the role of calcium-activated chloride channels in VNO neurons and rather offer a vague conclusion for their study (evidence for modulation of spike activity). This basically means that they invite the reader to make sense of it all. Being overly cautious in that way, they leave the story descriptive and incomplete. Moreover, they refrain from emphasizing the central points (why two channel types? Inhibitory or excitatory modulation?) and, instead, introduce speculative bits like the idea of heteromeric complexes of the two channel types, for which there is absolutely no evidence in VNO neurons. I think the Discussion needs to be re-written with the intent of helping the reader to appreciate the conceptual progress that was achieved in this very nice study.

Reviewer 2

In this manuscript, the authors use gene-knockout mouse models to investigate the individual contribution of two calcium-activated chloride channels, TMEM16A and TMEM16B, to physiological activities in vomeronasal sensory neurons (VSNs). Both TMEM16A and TMEM16B have previously reported being expressed in VSNs. The potential roles of these two channels in VSN sensory transduction and VSN physiological activities have also been explored in several studies, but the results are inconsistent among some studies. In this study, the authors confirmed the co-expression of both TMEM16A and TMEM16B in VSNs and their enriched localization to the microvillar layer in the vomeronasal epithelium. The authors also confirmed the loss of calcium-activated chloride current in TMEM16A-knockout VSNs but not in TMEM16B-knockout VSNs. By measuring spontaneous and urine-evoked spike activities, the authors show that TMEM16A, but not TMEM16B, has a role in modulating the spontaneous spike activity in VSNs and that both TMEM16A and TMEM16B contribute to modulating the firing patterns of evoked responses in VSNs.

Although the behavior significance of such influence of TMEM16A and TMEM16B on VSN physiology remains unclear, and it is still a mystery regarding how these two apically localized chloride channels influence the firing pattern but not transduction current, the detailed analyses of spontaneous and evoked activities of VSNs from TMEM16A and TMEM16B knockout mice described in this manuscript can be an important reference for future studies of peripheral coding of pheromones. The rationale of this study is sound, the experiments are well carried out, the data are of high quality, and the findings regarding the change in firing pattern in VSNs mutant mice are novel.

I do not have any major, nor minor, concerns regarding this manuscript, except in one place in the results, page 10 line 301, where the authors state, “we normalized the ISI distribution for each single VSN before adding it to create the ISI distribution ...”. It is not clear how the normalization is done. It's better to explain it further.

(The following is for a discussion point, and the authors need not address the questions in the revised manuscript. I am just curious about the answer to these questions.)

Do the authors know the relative expression levels of the two channels in VSNs? Could the differential contributions of the two channels to VSN physiology result from their relative expression levels? Also, an interesting finding in this study is that TMEM16A seems to exert a biphasic effect on VSN physiology. The authors show that TMEM16A differentially regulates spontaneous and evoked spike activities in VSNs -- positively regulates spontaneous activity, while negatively regulates evoked activity. What do these changes in firing pattern mean in terms of VSN function or pheromone coding? Does this biphasic regulation by TMEM16A benefit VSNs to allow higher sensitivity (as the result of higher spontaneous rate) and reduce potential saturation in instant spike firing?

## Author Response

To Dr. Christina Zelano, Reviewing Editor of eNeuro

Revision of the manuscript eN-NWR-0179-21 “TMEM16A and TMEM16B modulate pheromone-evoked action potential firing in mouse vomeronasal sensory neurons”.

We thank the Reviewing Editor and the two Reviewers for their careful reading and constructive comments on our manuscript. We believe that the revised manuscript has been improved by taking into account all the comments. We address point by point the specific comments below.

Synthesis Statement for Author (Required):

Both reviewers agreed that this is an interesting study that is a significant advancement in the field, as it provides the first high-quality data on the question of how pheromone-induced signals are modulated by calcium-dependent chloride currents ín pheromone-sensitive neurons. The reviewers had some important comments that can be addressed by adding some comments and explanations to the text. They also asked for some clarifications of the methods, which need to be added to the manuscript. Please see below the original comments from the reviewers:

Reviewer 1:

The authors present evidence for a modulatory role of calcium-activated chloride channels in signal generation by vomeronasal neurons in mice. The interest of this story lies in the fact that the output signals of sensory neurons are often finely-tuned by calcium-dependent ion channels, and there is growing evidence that TMEM16-type calcium-activated chloride channels serve such a purpose. For mouse VNO neurons, the authors show that the channels basically double interspike intervals, thus putting the brake on afferent signaling. Following a small series of papers on TMEM16 channels in VNO neurons, the present manuscript provides the hitherto most reliable data and is suited to provide a first plausible working hypothesis. While several aspects of this report require additional attention, the results are important for the fields of neuromodulation and sensory physiology.

We thank the Reviewer for the positive comments.

Points that need to be addressed in revision:

1. The authors suggest that two different TMEM16 ion channels operate in VNO neurons, a notion that is well founded by previous studies. According to literature, one of the main functional differences between the two is a 10-fold difference in calcium sensitivity, with concentrations for half-maximal activation of about 0.2 μM in TMEM16A and near 2 μM in TMEM16B. In their whole-cell experiments, the authors use a calculated (not measured) calcium concentration of 1.5 μM, which is expected to saturate TMEM16A, but cause little activation of TMEM16B. This approach is problematic as the real calcium concentration may differ substantially from the calculated one (3.029 mM CaCl + 10 mM HEDTA, pH 7.2), an uncertainty that all researchers face when working with cell calcium. In the present context, this point is of particular relevance because the “1.5 μM calcium” solution may indeed selectively activate TMEM16A, but not TMEM16B. This point must be made transparent for the reader. If, for example, whole-cell traces in Fig. 2 are similar in Tmem16b-ko and wild-type neurons, this does not indicate that TMEM16B is not expressed, but it may simply indicate that the calcium concentration within the pipette solution was not sufficiently high to activate TMEM16B. In fact, if the authors were interested in looking for TMEM16B currents, they should have used a different calcium concentration (10 μM) in an additional, separate set of recordings. That would have been wise, as all the interesting effects of the chloride channels reported later in the manuscript could be explained with two channel types of different calcium sensitivities.

We agree with the Reviewer that buffering Ca2+ in the micromolar range is very difficult. We used precisely the same solution composition (10 mM HEDTA, 3.209 mM CaCl2, pH 7.2) that was previously experimentally measured and found to correspond to 1.5 μM free Ca2+. We added this in the Methods section (lines 159-164): “The 1.5 μM free Ca2+ solution was obtained by adding 3.209 mM CaCl2. The added concentration was the same as that previously used to obtain 1.5 μM free Ca2+ experimentally determined by Fura-4F (Molecular Probes-Invitrogen) measurements by using a LS-50B luminescence spectrophotometer (PerkinElmer, Wellesley, MA; Pifferi et al., 2006, 2009a, 2009b) and based on calculation with the program WinMAXC (C. Patton, Stanford University, Stanford, CA; Patton et al., 2004).”

We also agree with the Reviewer that a higher Ca2+ concentration would have been better to activate TMEM16B. Indeed, we tried to use 13 and 100 μM Ca2+, but VSNs became very leaky with these solutions. We added the following lines to the Discussion section (lines 418-432): “Indeed, although TMEM16B is less sensitive to Ca2+ than TMEM16A, it should be activated by 1.5 μM Ca2+ at +100 mV, as an EC50 of ∼2 μM has been reported at high voltages (Cenedese at al., 2012; Adomaviciene at al., 2013). We also attempted to increase intracellular Ca2+ to 13 and 100 μM, respectively, but VSNs in slices became leaky with these solutions. However, previous experiments in isolated VSNs successfully increased intracellular Ca2+ up to 2 mM without finding any measurable Ca2+-activated chloride current in VSNs from Tmem16a cKO mice (Amjad et al., 2015). Yet, as the average whole-cell leak current at +100 mV was ∼300 pA, we cannot exclude that we missed a small current activated by TMEM16B. Lack of current in Tmem16a cKO mice was also measured by Münch et al. (2018) who suggested the possibility that Ca2+ concentration in the microvilli does not reach the concentration necessary to activate TMEM16B”.

2. The control recordings to determine possible changes of input resistance and resting membane potential at zero calcium are well done but lack, to my understanding, one aspect. TMEM16A was reported to open at positive voltages even in calcium-free solution. The voltage-clamp recordings in Fig. 3 show that this does not alter passive properties up to +40 mV, a voltage range where AP dynamics can conceivably be modulated by chloride currents. The authors demonstrate that this is not the case and may consider attracting the reader´s attention to this point.

Thanks for this suggestion, we added (lines 277-280) “in the range from -100 up to +40 mV. As TMEM16A can also be activated by high positive voltages in absence of intracellular Ca2+ (Xiao et al., 2011; Segura-Covarrubias et al., 2021), these results show that TMEM16A does not alter passive properties at membrane potentials up to +40 mV”.

3. To check whether glomerular targeting in Tmem16a-ko mice was disturbed, the authors crossed two mouse lines to obtain V2R1b-GFP-TMEM16A-cKO animals. To assess the validity of these experiments, readers need to know which generation of cross-breeding was used. In particular, was this line heterozygous with respect to Tmem16a so that one intact Tmem16a allele was present? In that case, the lack of effect on OE/OB wiring could be explained by the remaining allele.

We added in the results section this information (lines 329-331): “V2R1b-GFP-TMEM16A cKO mice were homozygous for V2R1b-GFP and Tmem16a-Flox genes, and heterozygous for OMP/Cre, while control animals were homozygous for V2R1b-GFP, Tmem16a-Flox and OMP genes, lacking the expression of Cre recombinase.”

4. A strength of this study was that the authors selected viable, responsive VNO neurons for examination, discarding all cells that were either unresponsive or did not fit control criteria (like the lack of response to artificial urine). The success of the study clearly justifies that strategy. The authors may consider adding to the Discussion a statement on how they assess the possibility that such a strict selection procedure can introduce a bias to the outcome of the study.

We added in the Discussion section this information (lines 507-511): “It is important to note that our criteria for selecting neurons responding to urine are more stringent with respect to those of other studies (Münch et al., 2018; Kim et al., 2011) and could have excluded some VSNs with very high spontaneous firing frequencies. We argue that our method, similar to that used by Arnson and Holy (2011), is suitable to limit the analysis to a subset of clearly viable neurons.”

5. The authors shy away from specifying a distinct working hypothesis on the role of calcium-activated chloride channels in VNO neurons and rather offer a vague conclusion for their study (evidence for modulation of spike activity). This basically means that they invite the reader to make sense of it all. Being overly cautious in that way, they leave the story descriptive and incomplete. Moreover, they refrain from emphasizing the central points (why two channel types? Inhibitory or excitatory modulation?) and, instead, introduce speculative bits like the idea of heteromeric complexes of the two channel types, for which there is absolutely no evidence in VNO neurons. I think the Discussion needs to be re-written with the intent of helping the reader to appreciate the conceptual progress that was achieved in this very nice study.

We rewrote the Discussion with the intent of helping the reader to appreciate the progress, as suggested by the Reviewer.

Reviewer 2

In this manuscript, the authors use gene-knockout mouse models to investigate the individual contribution of two calcium-activated chloride channels, TMEM16A and TMEM16B, to physiological activities in vomeronasal sensory neurons (VSNs). Both TMEM16A and TMEM16B have previously reported being expressed in VSNs. The potential roles of these two channels in VSN sensory transduction and VSN physiological activities have also been explored in several studies, but the results are inconsistent among some studies. In this study, the authors confirmed the co-expression of both TMEM16A and TMEM16B in VSNs and their enriched localization to the microvillar layer in the vomeronasal epithelium. The authors also confirmed the loss of calcium-activated chloride current in TMEM16A-knockout VSNs but not in TMEM16B-knockout VSNs. By measuring spontaneous and urine-evoked spike activities, the authors show that TMEM16A, but not TMEM16B, has a role in modulating the spontaneous spike activity in VSNs and that both TMEM16A and TMEM16B contribute to modulating the firing patterns of evoked responses in VSNs.

Although the behavior significance of such influence of TMEM16A and TMEM16B on VSN physiology remains unclear, and it is still a mystery regarding how these two apically localized chloride channels influence the firing pattern but not transduction current, the detailed analyses of spontaneous and evoked activities of VSNs from TMEM16A and TMEM16B knockout mice described in this manuscript can be an important reference for future studies of peripheral coding of pheromones. The rationale of this study is sound, the experiments are well carried out, the data are of high quality, and the findings regarding the change in firing pattern in VSNs mutant mice are novel.

We thank the Reviewer for the positive comments.

I do not have any major, nor minor, concerns regarding this manuscript, except in one place in the results, page 10 line 301, where the authors state, “we normalized the ISI distribution for each single VSN before adding it to create the ISI distribution ...”. It is not clear how the normalization is done. It's better to explain it further.

We added (lines 304-306): “we normalized the ISI distribution by dividing the number of spikes for each bin by the total number of spikes for each single VSN before adding it to create the ISI distribution for each genotype”.

(The following is for a discussion point, and the authors need not address the questions in the revised manuscript. I am just curious about the answer to these questions.) Do the authors know the relative expression levels of the two channels in VSNs?

We rewrote the Discussion as suggested by Reviewer 1 and also included answers to the questions of Reviewer 2. About the relative expression of the two channels, we added (lines 409-413): “The relative expression level of the two channels has been evaluated in the transcriptomic study of Ibarra-Soria et al. (2014). They found high expression for both transcripts, with Tmem16a levels about twice that of Tmem16b, without significant difference between males and females (Dataset S1 and Dataset S3 in Ibarra-Soria et al., 2014).

Could the differential contributions of the two channels to VSN physiology result from their relative expression levels?

It is likely that different contribution of the two channels in VSNs is due to a different expression levels, as well as to a different calcium sensitivity and/or different isoforms expressed in VSNs. We added (lines 466-472): “As TMEM16A has a higher Ca2+ sensitivity than TMEM16B, a possible explanation for the lack of contribution of TMEM16B in VSNs is that the spontaneous activation of the signal transduction cascade leads to intracellular Ca2+ increase, which is insufficient to activate TMEM16B. Moreover, Tmem16b transcript expression level has been reported to be half of that of Tmem16a in VSNs (Ibarria-Soria et al., 2014) indicating that TMEM16B, in addition to being less sensitive to Ca2+, may also have a lower expression than TMEM16A”.

Also, an interesting finding in this study is that TMEM16A seems to exert a biphasic effect on VSN physiology. The authors show that TMEM16A differentially regulates spontaneous and evoked spike activities in VSNs -- positively regulates spontaneous activity, while negatively regulates evoked activity. What do these changes in firing pattern mean in terms of VSN function or pheromone coding? Does this biphasic regulation by TMEM16A benefit VSNs to allow higher sensitivity (as the result of higher spontaneous rate) and reduce potential saturation in instant spike firing?

We rewrote the Discussion, following also the suggestion of Reviewer 1, to better explain the different effects of Tmem16a and Tmem16b deletion on spontaneous and evoked activity.
